# No more single stories: The case of global adolescent pregnancy care

**DOI:** 10.1371/journal.pgph.0006088

**Published:** 2026-03-06

**Authors:** Farnaz Sabet, George C. Patton, Dominique P. Béhague, Susan M. Sawyer, Oloruntomiwa I. Oyetunde, Audrey Prost

**Affiliations:** 1 Centre for Adolescent Health, Royal Children's Hospital, Parkville, Victoria, Australia; 2 Murdoch Children's Research Institute, Parkville, Victoria, Australia; 3 Department of Paediatrics, The University of Melbourne, Parkville, Victoria, Australia; 4 Vanderbilt University, Nashville, Tennessee, United States of America; 5 King's College London, London, United Kingdom; 6 Institute of Child Health, College of Medicine, University of Ibadan, Ibadan, Nigeria; 7 Institute for Global Health, University College London, London, United Kingdom; Nagasaki University, JAPAN

## Introduction

Effective implementation of health interventions is complex, particularly in resource-constrained settings. Historical and socio-political processes influence the framing and prioritisation of health issues as well as approaches to clinical care and interventions [1,2].

One group that finds itself near the bottom of the power matrix of almost every society is pregnant adolescents. Young and female, often poor, from discriminated racial and ethnic groups in marginalised and isolated communities [3], the vast majority of pregnant adolescents live in low- and middle-income countries (LMIC) [4]. Though taking on new adult responsibilities, they are often subject to others making decisions on their behalf, and are excluded from many democratic processes.

In this essay, we use the example of care for pregnant girls to provide insights into the consequences of adopting one dominant framing when responding to complex social and health conditions. We draw on a published systematic review of interventions for pregnant adolescents in LMIC [3], which found the evidence on interventions to support these girls through their pregnancy was scarce. The findings were particularly surprising given the large amount of literature on poorer outcomes associated with adolescent pregnancy. Systematic reviews can expose evidence gaps, but they often do not analyse why the evidence is missing. This essay argues that a dominant framing adopted from the Global North, that adolescent pregnancy is a public health problem only in need of prevention, has contributed to the neglect of high-quality care for pregnant girls.

This essay does not minimise the need for and importance of early pregnancy prevention programmes, rather we advocate for a clearer consideration of social power in the production, credibility, use and distribution of knowledge [5], so that knowledge production and programmes also respond to the needs of the 21 million adolescents who become pregnant each year [6]. This argument is supported by drawing on evidence from Latin America, where local scholarship has encouraged the development of alternative framings of adolescent pregnancy which better support responsive interventions for pregnant adolescents. In conclusion, we underscore the importance of local scholarship for understanding and addressing complex and diverse health experiences.

## Adolescent pregnancy is framed as a problem needing prevention

We conducted the only systematic review of interventions addressing any health outcome for pregnant adolescents in LMIC, defined as girls aged 10–19 years at any gestation and up to six weeks postpartum, and found a surprising lack of evidence [3]. We conducted extensive searches for interventions with evidence, including quantitative or qualitative studies regardless of quality, on any aspect of perinatal care for adolescents and their newborns in LMIC. We full text screened maternal health studies to see if they included adolescents and also sub-analysed for effects in them. Quantitative articles required at least a before-and-after comparison or have a comparator group and descriptive accounts were excluded. No language restriction was applied. A broad search strategy was conducted across 16 databases, and we additionally contacted many experts and organisations. Despite screening over 30 000 articles, we found surprisingly little evidence for improving outcomes for adolescents and their newborns over twenty years across all aspects of antenatal, delivery, post-natal and newborn care. Most of the 62 studies had low quality evidence; only three quantitative studies at low risk of bias were identified and 12 qualitative studies had pertinent insights. Only 35 of the studies focused specifically on pregnant adolescents. Interventions ranged from micronutrient supplementation, to improving maternal health knowledge and literacy, to conditional cash transfer programs.

Rather than consider the unique needs of this age group, many studies in the review generally framed adolescent pregnancy as a problem that should not exist. Maternal health researchers rarely disaggregated their findings by the typical age groups that identify adolescents, and most interventions did not treat pregnant adolescents differently to pregnant adults. In trials such as those on nutritional supplementation in pregnancy, some differential effects in adolescents compared to adults were seen, but these sub-analyses were not prespecified and the findings seemed almost serendipitous [7–9]. The few trials that did pre-specify subgroup analyses in girls aged 15–19 years [10,11] did not adapt any aspect of the intervention for them. This is despite a wealth of literature highlighting poorer outcomes in pregnant adolescents and their newborns [12]. When pregnant adolescents were recognised and included, they were often framed as an at-risk group with programmatic focus on reducing sexual risk rather than providing pregnancy care [13].

Our review highlighted the extreme evidence gap in relation to adolescent maternal health. It recommended more research on adolescent-specific needs in maternal care and that maternal health studies in settings with a significant proportion of pregnant adolescents should recruit them and analyse for effects in them. Despite these important findings, the review did not explore *why* there has been a relative neglect of interventions to support and improve outcomes for pregnant adolescents.

To answer this question requires us to unpack the dominant framing on adolescent pregnancy which stems from the Global North. The increased data on adverse consequences for mother and child coupled with substantial cultural shifts in the Global North helped to frame adolescent pregnancy as primarily a preventative issue from the 1970s onwards [14]. As researchers have shown, this shift is linked to the well documented rise of neoliberal values in reproductive health policy and public health more generally [15]. Neoliberalism in this literature refers to approaches that tend to view health improvements as, first and foremost, an individual responsibility. The individual is primarily responsible for their health and well-being and morally responsible for rational action to protect their own health with comparatively less attention paid to the broader environments that ensures equitable access to services and opportunities [16,17].

Pregnant girls were subsequently increasingly framed as lacking in health knowledge, careless in their behaviour or as victims who were nonetheless personally responsible for becoming pregnant [15]. In 1995, in his State of Union address, President Clinton referred to unmarried teen pregnancies and births as “our most serious social problem.” [18] The desirable path for a young person has become one of pursuing educational and economic opportunities [14], which is assumed to be incompatible with motherhood. While sexual health policies commonly encourage young people to take control of their sexuality and reproduction [15], an unintended consequence is that stronger neoliberal ideologies can be associated with less sympathy for and less positive attitudes about girls who do become pregnant, particularly if the pregnancy was unintended [19].

Within this framing, the predominating policy response, presumed to be globally applicable, has been to focus on preventing teenage pregnancy, with much less emphasis on supporting adolescent parents and their children. This framing, which emphasises individual choice and responsibility, ignores diverse cultural contexts as well as the structural and often intersecting disadvantages that many teenage mothers face, including gender, age, race and poverty [14]. It also oversimplifies the complex relationship between adolescent pregnancy and poorer health and developmental outcomes, which may be due to multiple factors associated with social disadvantage [20,21].

Clinical and public health emphasis on the prevention of pregnancy is vital, but it cannot be the only response leaving millions of girls who do become pregnant without high-quality care. This narrative – a single story [22] – that originated from the Global North ignores the enormous global diversity of adolescent pregnancy experiences. Can the same narrative in settings as different as Iran and Ghana be truly responsive to the complex experience of adolescent pregnancy and lead to effective interventions? Our review did identify one region that has adopted a more flexible, context specific approach to understanding adolescent pregnancy – Latin America. There we saw glimpses of an alternative and more promising approach, where local scholarship led to very different narratives about adolescent pregnancy.

## An alternative framing from Latin America

A large proportion of the studies in our systematic review came from Latin America (46%) [3]. Many were published in Spanish or Portuguese in local journals and databases, and gave insights into framings not found in the Anglophone literature. Most of these studies were conducted by academics at public universities using local funding and employed qualitative or mixed methods approaches that captured adolescent perspectives. Among the 22 studies from this region, 13 offered an alternative to the dominant framing of pregnancy prevention seen elsewhere, with interventions striving to respond to pregnant girls’ needs. These needs were not defined by health professionals or community leaders. Rather, researchers used inductive methods to enable pregnant adolescents to define their own needs. Interaction with the health system was not framed as a service given to at-risk, uneducated, and vulnerable pregnant adolescents, but rather a privileged space with potential for “construction of collective and individual significance about pregnancy and teenage motherhood.” [23]^(p1)^

Changing how health issues are framed can change the types of interventions offered and how these are experienced by recipients and evaluated. One intervention offered pregnant adolescents individualised, well thought out nutrition consultations interspersed with three group sessions [24]. The intervention had no effect on reducing gestational weight gain, but in their mixed methods evaluation the researchers did not place blame on the girls for failing to respond to the program, nor did they use this failure to reinforce the narrative that the only response to adolescent pregnancy is to prevent it. Rather they reflected on the program quality itself. The researchers viewed these girls as adolescents with valid needs and noted the mismatch between the technical nutrition guidelines and the girls’ aspirations. Adolescents saw nutrition guidelines as a restriction to their freedom. As the authors concluded, this focus on the technicalities of nutrition guidelines rather than the concerns of adolescents and their food cultures, led the adolescents to become annoyed and impatient with consultations, ultimately “denying them the possibility of consciously participating in the decision-making process about their nutritional care.” [24]^(p. 799)^

When adolescents were framed as partners in their own health care with legitimate needs, evaluation by researchers also became more perceptive of when pregnant adolescents were being disrespected. Another study investigated the relationship between health professionals and adolescents in a public hospital programme for pregnant adolescents [23]. Despite the hospital unit focusing on adolescent maternal care, relationships were characterised by power imbalance, with expectations of subjugation and obedience. As one health professional is quoted stating, “I am very democratic, as long as they do what I want." [23]^(p.778)^ When pregnant adolescents are framed as irresponsible, their lack of engagement with health services is blamed on their individual behaviour and attitudes, shifting attention from the quality of health care they receive. This study’s alternative framing of pregnant adolescents as legitimate users of health care led to a broader evaluation of clinic services beyond personal responsibility, identifying poor health worker engagement as a key factor impacting pregnant girls’ engagement with health services.

## Local scholarship is needed to inform responsive framing

Latin American scholarship has also questioned the construction of adolescent pregnancy as a public health crisis [25 ^–^27]. Durán and colleagues [25], for example, provide historical and legal perspectives on the construction of adolescent pregnancy in Argentina. They propose that the introduction of compulsory schooling turned adolescent pregnancy into an obstacle to progress, creating a perception of pregnant teenagers as deviant. Moreover, they describe South American stereotypes of motherhood which tend to denigrate single motherhood and advocate an adult-centric approach. The authors argue that young mothers should drive their own interventions and be seen as active subjects and defenders of their rights as citizens. In another piece, Heilborn and colleagues [26] challenge the now dominant mainstream Brazilian perspective that equates teenage pregnancy with poverty, illegitimate births, and deviation from ideal educational pathways and subsequent gains, highlighting that experiences of young people, including pregnant girls, are much more nuanced and diverse.

These critical viewpoints are outgrowths, in part, of the Latin American Social Medicine (LASM) school of thought that emerged in the 1970s and still influences public health in the region and beyond. LASM draws on experts based mainly in public institutions, many of whom work synergistically with activists and groups engaged in political transformation [28]. It highlights upstream determinants of health and pursues core principles of health justice, often based on Marxist traditions. It links theory and practice to promote practices with clear ideological objectives [29] that defend health as a citizen’s right and a duty of the state. LASM has a distinctive methodological tradition that critiques and analyses what is usually presented as neutral technical knowledge [29]. The links between research, social activism, and political engagement in LASM have likely contributed to creating an academic environment as well as a body of knowledge that has fostered alternative narratives of adolescent pregnancy.

Importantly, a key strength in LASM scholarship is the inclusion of a science studies perspective that investigates how research is not value-neutral but is, rather, interwoven with social, moral, and political values, contexts, and biases [30]. Building on this tradition, for instance, longitudinal research conducted within the 1982 Pelotas (Brazil) Birth cohort, which tracked some 6000 infants across health, socioeconomic and developmental trends through to adulthood [31], has questioned the commonly held assumption that teen pregnancy is a primary driver of school dropout rates. Instead, this research suggests that young women's sense of alienation from education often predates and may even play a role in teenage pregnancies [32]. One of us (DB) conducted additional research with that cohort to show that a key contributor to young women’s alienation from education was the experience of being stigmatised and stereotyped by teachers and school staff through health education campaigns. These campaigns were so intensely centered on preventing pregnancy that teens, especially poor and racialized teens, recounted feeling singularly defined as psychologically immature and prone to sexual risk-taking [27,33].

Although the dominant framing of adolescent pregnancy as one of risk and vulnerability is gaining prominence in Latin America, regional scholarship is consistently showing how this framing diverts policy attention from underlying social and economic inequities [34]. In doing so, these scholars have proposed different ways of conceptualising interventions for pregnant adolescents. For example participatory group settings [35] and home visits [36] for maternal care that change power dynamics seen in clinic settings, and encourage participation of pregnant adolescents, and would arguably, better responds to their needs [37].

## Moving beyond dominant frameworks and advancing rigorous regional and local scholarship

The purpose of this viewpoint is not to romanticise one response to adolescent pregnancy or to criticise another. Rather, we wish to highlight the need to move beyond one dominant global framing for this complex condition which has contributed to the neglect of pregnant adolescent care. Adolescent pregnancy and motherhood are culturally and socially heterogenous phenomena and require local scholarship that builds on local bodies of knowledge to produce appropriate responses.

Local scholarship goes beyond stakeholder consultations or adopting participatory methods for interventions. It entails interdisciplinary locally grounded scholarship that has been invested in and developed over years. Alternative narratives on adolescent pregnancy in Latin America did not result from local engagement of diverse stakeholders for discrete research projects but were fed by longstanding local and regional scholarship in disciplines such as anthropology, religion and politics amongst others. Do we as a global health community appreciate and draw on local scholarship and intellectuals, often in languages other than English, to inform and challenge our thinking around complex issues? Do we advocate for and understand the need to advance scholarship at public universities and other institutions globally, across diverse disciplines not only health?

The movement for epistemic justice in global health sits at the forefront of advocating for the development of local and regional scholarship. It involves multiple actors who aim to identify and correct imbalances in power, including reversing the immediate legacies of colonialism [38]. Bakhuni and Abimbola call out the implicit biases and prejudices towards knowledge production in academic global health that favour dominant groups [39]. These biases interfere with the ability of marginalised groups to produce knowledge, use it and circulate it, and lead to ascribing lower credibility to knowledge that is shared by such groups or individuals. The path forward is not straightforward but complex and uncertain. It is often addressed as an issue of exclusion, and the response thus is to include or amplify marginalised voices [40]. This is insufficient. As highlighted in the case of Latin America, a local and rigorous body of scholarship that is often facilitated by publicly funded infrastructure is required. This scholarship is critical for informing the development and particularly the framing of responsive interventions.

**Fig 1:**
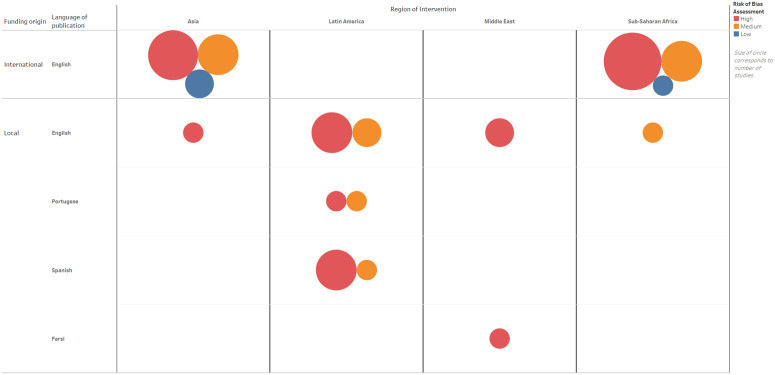
Publication funding and language by region for interventions for pregnant adolescents in Low and Middle-Income Countries.

Supporting local and regional scholarship is challenging. The breakdown of funding and language across the articles in our review of interventions for pregnant adolescents (Fig 1) show the influence of the Global North in producing research that is technically assessed as ‘low risk of bias’. Many of the studies from Latin America were funded and conducted locally, and not translated into English. They were also generally deemed to be ‘of poor quality’ and at ‘high risk of bias’ according to quality assessment tools recommended for biomedical reviews. This contrasted with studies from other regions, where most local researchers were affiliated with academics and universities in the Global North, had obtained international funding, published in English, and were conversant with the requirements of quality assessment tools, yet invariably adopted the same overarching framing of empowering individual autonomy and choice to prevent pregnancy. Although systematic reviews intend to synthesise the best evidence on a topic, and risk of bias assessments are needed, when assessing complex health interventions across diverse global health settings, there is also a need to consider how that evidence is framed.

Not identifying local scholarship from other regions can also stem from limited exposure to this work. Many LMIC researchers have reduced global exposure to their finding and thinking due to multiple barriers for publication, including cost. Most of the Latin American studies adopting different framings were identified through the Latin-American and Caribbean System on Health Sciences Information (LILACS), an open access comprehensive index of scientific and technical literature from the Latin-American and Caribbean region. Although similar databases exist in other regions, they are of varying quality and functionality. Developing these databases further by WHO and enhancing their reputation and use, will assist researchers and importantly policy makers from LMIC to easily access local research.

## Conclusion

The dominant framing of adolescent pregnancy emphasises that the ‘right’ individual ‘choice’ is to not get pregnant, which has led to neglect of quality adolescent maternal health care provision. Pregnant adolescents are generally viewed as either a vulnerable and at-risk group, not recognised at all, or assumed to have the same needs as adults. These approaches patronise pregnant adolescents and deny them active partnership in the development of policies and programmes to address needs that they voice.

We contend that this is a fundamental issue of epistemic injustice where neoliberal framings, emphasising individual choice and responsibility [1] have come to dominate global policy, impacting the generation of knowledge that could respond better to local contexts. This argument is further strengthened with the example from Latin America where local scholarship has supported alternative framings of pregnancy in adolescents, as a nuanced and diverse experience. This framing led to unique and responsive evaluations and interventions which placed broader responsibility on social factors and the health system rather than individual girls by, for example, ensuring that health care workers reflect on how power imbalances in the patient-provider relationship can alienate pregnant adolescents from the health care system.

Using the example of adolescent pregnancy care, we propose that moving towards epistemic justice requires investment in diverse and rigorous local scholarship, as well as acceptance of alternative approaches that may not tow the dominant narrative line. This will benefit from locally funded research led by local research institutions and will benefit from further development of open-access research databases in languages other than English. We also need to more explicitly recognise how interventions have been framed, and by whom, so that we can prioritise framings based on local rigorous scholarship that allows more critical roles for people, in this case pregnant adolescents, to frame issues of concern to them.
